# Dendritic Cell-Derived Exosomes: Next Generation of Cancer Immunotherapy

**DOI:** 10.3390/biomedicines13102497

**Published:** 2025-10-14

**Authors:** Rajib Dhar, Swarup Sonar, Asmit Das, Nur Aliaa Sorfina Tajul Akmal, Ainil Hawa Jasni, Vinod RMT Balasubramaniam, Kumaran Narayanan, Vetriselvan Subramaniyan

**Affiliations:** 1 Division of Pharmacology, Sir Jeffrey Cheah Sunway Medical School, Faculty of Medical and Life Sciences, Sunway University, Bandar Sunway 47500, Selangor Darul Ehsan, Malaysia; 2 Department of Oncology, Neuron Institute of Applied Research, Amravati 444601, Maharashtra, India; 3 Universiti Kuala Lumpur Royal College of Medicine Perak (UniKL RCMP), 3, Jalan Greentown, Ipoh 30450, Perak, Malaysia; 4 Jeffrey Cheah School of Medicine and Health Sciences, Monash University Malaysia, Bandar Sunway, Subang Jaya 47500, Selangor, Malaysia

**Keywords:** cancer, metastasis, DC exosomes, cancer immunotherapy

## Abstract

Dendritic cells (DCs) are the most highlighted cell population for cancer immunotherapy development. Currently, DC-derived exosomes show promising anti-cancer activity. Exosomes are a subpopulation of extracellular vesicles (EVs) and originate from endosomes. It transports dynamic molecular cargos such as DNA, RNA, protein, and lipid. This cellular cargo exchange reprograms the recipient cell naturally. In cancer research, DC-derived exosomes (DEXs) are used as a therapeutic tool. There are some approaches followed in the application of DEX in cancer as a therapeutic tool. DEX-based drug delivery, tumor antigen-loaded DEX, and modified DEX are applicable approaches in cancer therapy. DEXs are biocompatible, nontoxic, and have ability-specific targeting. On the other hand, this method faces some challenges, such as large-scale production, isolation, and heterogeneity. A multidisciplinary approach (advanced nanotechnology, multi-omics, and single-exosome profiling) comes up with a solution to this issue. This review provides a comprehensive overview of the DEX approach, tracing its developmental journey and therapeutic application in cancer immunotherapy. It examines key findings from clinical trials and outlines the challenges and future research directions in this field, ultimately underscoring the potential of DC-derived exosomes as a research-backed, cell-free solution for the next generation of cancer immunotherapies.

## 1. Introduction

**Figure 1:**
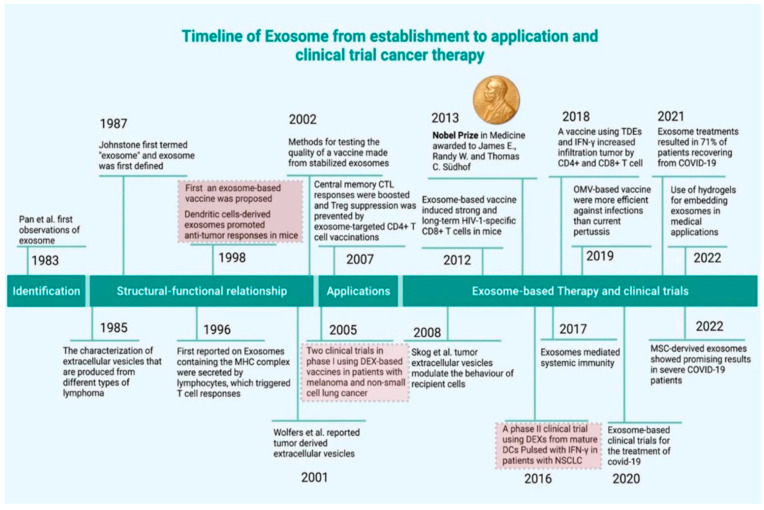
Exosome-based cancer therapy (reproduced with permission under Creative Commons CC BY 4.0 license from ref. [33] Copyright 2022, Springer Nature).

Cancer remains one of the major causes of mortality and represents a notable public health challenge globally, despite substantial advancements in conventional treatment modalities such as surgery, chemotherapy, and radiotherapy [1,2]. Over the past two decades, immunotherapy has emerged as a transformative approach in oncology, utilizing the body’s innate and adaptive immune responses to recognize and destroy malignant cells by inhibiting oncogenic pathways [3,4]. Major approaches include immune checkpoint inhibitors (such as anti-PD-1, PD-L1, CTLA-4) that enhance T-cell responses, and adoptive cell therapy, like CAR T-cell therapy, monoclonal antibodies, and therapeutic vaccines [4]. Though the concept of cancer vaccines dates back to the 1970s, challenges such as immune escape and poor immunogenicity have hindered the progress of cancer vaccines in clinical translation [5,6]. Therapeutic cancer vaccines offer a wide range of approaches, including subunit vaccines (peptides, proteins, and tumor lysates), neoantigen-based personalized vaccines, dendritic cell-based vaccines, nanoparticle-assisted lymph node-targeting systems, mucosal and intratumoral delivery platforms, scaffold-based and injectable depot vaccines, and in situ tumor vaccines, all of which are designed to address tumor immune tolerance, enhance T cell priming, and improve tumor-specific immune responses [7,8,9]. Moreover, prophylactic vaccines such as the Human Papillomavirus (HPV)-based vaccines (available in bivalent, quadrivalent, and nonavalent forms) have shown significant success in preventing HPV-associated malignancies, particularly cervical cancer. However, these vaccines are preventive in nature and offer protection only against select HPV types, with limited therapeutic benefit once infection or malignant transformation has occurred [10]. This underlines the need for more effective broad-spectrum therapeutic strategies. Among the various immunotherapeutic strategies, dendritic cell (DC)-based vaccines have gained significant attention due to their pivotal role as professional antigen-presenting cells (APCs) that initiate and regulate tumor-specific immune responses. However, despite promising preclinical outcomes, the clinical efficacy of DC vaccines has been hindered by several limitations and challenges related to their ex vivo manipulation, limited in vivo migration, and immunosuppressive tumor microenvironment (TME) [11]. Addressing these challenges, recent research has shifted towards dendritic cell-derived exosomes (DEXs) as a promising alternative cell-free approach that could overcome many of the hurdles and push the boundaries associated with whole-cell DC therapies [6,12,13]. Exosomes are naturally secreted, nano-scale (30–200 nm) extracellular vesicles (EVs) of endosomal origin that are released by a wide variety of cells. They facilitate intercellular communication by transporting bioactive molecular cargoes like proteins, lipids, and nucleic acids (mRNAs, miRNAs, lncRNAs, and DNA fragments) [6,14,15]. Exosomes can be derived from a diversity of cells, including tumor cells [16], mesenchymal stem cells [17], and immune cells [18]. Recent scientific advances even suggest that plant-derived exosomes [19] and milk-derived exosomes [20] hold promising potential for cancer therapy and for minimizing drug resistance. Particularly, DC-derived exosomes (DEXs) preserve essential immunostimulatory properties of their parental dendritic cells. These properties include the presence of major histocompatibility complex (MHC) class I and class II molecules, costimulatory molecules (such as CD80, CD86), and adhesion molecules (e.g., ICAM-1), making them highly competent in antigen presentation. Most importantly, DEXs can be loaded with tumor-associated antigens (TAAs) and engineered to enhance their immunogenicity, thereby acting as cell-free nanocarriers that activate T cells and natural killer (NK) cells to initiate robust anti-tumor responses. On top of that, their miniature size, morphological stability, biocompatibility, low toxicity, and significant ability to circulate biomolecules systemically support their potential for therapeutic applications in cancer immunotherapy [13,21,22]. The immunomodulatory functions of DEXs have been extensively explored in preclinical cancer models, demonstrating their ability to stimulate CD8+ cytotoxic T lymphocytes (CTLs), polarize Th1 responses, and synergise with immune checkpoint inhibitors [13,23,24]. Several studies have also demonstrated that DEXs can modulate the tumor microenvironment by enhancing immune cell infiltration, reprogramming tumor-associated macrophages, and inhibiting regulatory T cells (Tregs), thus shifting the TME from an immunosuppressive to an immunostimulatory state [12,23]. Notably, early-phase (Phase I) clinical trials have provided encouraging evidence on the safety, tolerability, and partial immunogenic efficacy of DEXs in patients with non-small cell lung cancer (NSCLC) and melanoma. These findings support the potential of DEXs as scalable, off-the-shelf immunotherapeutics in personalized cancer medicine [25,26]. Despite their tremendous promise, the clinical translation of DEXs still faces multiple hurdles. These include the need for standardized methods for isolation and purification, refined strategies for antigen loading, and scalable exosome production that meets general Good Manufacturing Practices (GMP) practices. Furthermore, improving in vivo targeting efficiency remains a key challenge [27,28]. Moreover, understanding the biodistribution, pharmacokinetics, and long-term immunological effects of exosome-based therapies is essential for advancing their clinical application. Advances in nanotechnology, molecular engineering, and systems immunology are expected to play a critical role in refining DEXs-based immunotherapies for enhanced specificity, durability, and efficacy [27,28]. As shown in Figure 1, the development of DEXs is described in detail in terms of application and clinical trials. DEXs represent a distinctive class of extracellular vesicles with several advantages over exosomes originating from tumor cells or mesenchymal stem cells (MSCs). Tumor-derived vesicles, while reflective of their cellular origin, often carry oncogenic proteins and nucleic acids that contribute to tumorigenesis and metastasis, thereby raising significant safety concerns for therapeutic application. By contrast, DEXs are enriched in immune-stimulatory molecules, including major histocompatibility complex (MHC) and co-stimulatory proteins, which collectively promote antigen-specific T-cell activation. This immunogenic profile positions DEXs as a safer and more effective option in immunotherapy [28]. MSC-derived exosomes have been extensively investigated for their regenerative and immunomodulatory properties, demonstrating utility in tissue repair and inflammatory disorders. However, their broad immunosuppressive effects may counteract the desired anti-tumor immune responses in cancer settings. From a translational perspective, MSC-derived exosomes benefit from scalability due to the robust expansion capacity of stem cells, which facilitates large-scale production [29,30,31]. Nevertheless, ongoing improvements in dendritic cell culture systems and standardized isolation methods are progressively addressing scalability challenges associated with DEXs [32]. Collectively, these comparisons underscore the unique positioning of DEXs as a therapeutic platform that integrates safety with potent immune activation, while maintaining growing feasibility for clinical translation. As research advances, DEXs are increasingly recognized as offering a balanced and clinically relevant alternative within the broader landscape of exosome-based therapies [32].

This review aims to provide a comprehensive overview of the current landscape of dendritic cell-derived exosomes in cancer immunotherapy, highlighting their biogenesis, functional mechanisms, engineering strategies, preclinical evidence, and translational potential. We also address the challenges and future directions that will shape the next generation of exosome-based cancer immunotherapies. By integrating the current knowledge and transformative insights, this review underscores the pivotal role of DEXs in reshaping the landscape of cancer immunotherapy and advancing toward clinically viable, cell-free immune therapeutics.

## 2. Biogenesis of Exosomes

**Figure 2:**
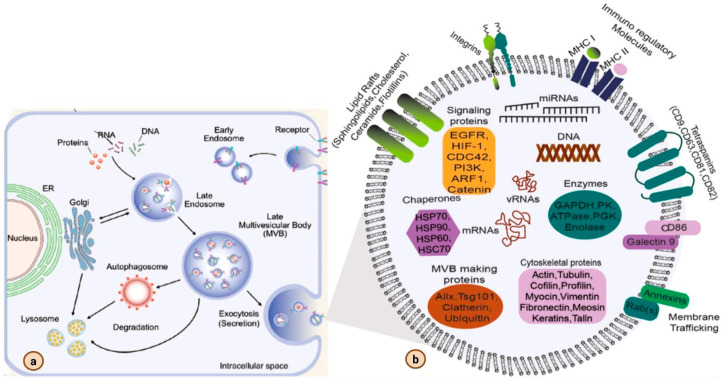
Exosome biogenesis and its components. (**a**) exosome biogenesis, and (**b**) exosome molecular cargo. (Adapted with permission from ref. [64] Copyright @ 2021 American Chemical Society.)

Exosome biogenesis (Figure 2) in animal cells is a sophisticated and highly coordinated process, fundamental to how cells communicate and maintain their internal balance. This journey begins deep within the cell, specifically within the endocytic pathway, where tiny internal pockets, known as intraluminal vesicles (ILVs), form inside larger compartments called multivesicular bodies (MVBs) [34,35,36]. These MVBs, once docked and fused to the plasma membrane, the cell’s outer membrane, release their ILV cargo as exosomes into the extracellular space to mediate cell-to-cell communication [34,35,37]. This biogenesis process is mainly regulated by two molecular mechanisms, the ESCRT-dependent and ESCRT-independent mechanisms [36,37,38,39,40]. The ESCRT-dependent mechanism is a highly regulated and orderly process that involves four protein complexes called the Endosomal Sorting Complexes Required for Transport (ESCRT), which function along with the VPS4 ATPase [34,41,42,43]. This process begins when ESCRT-0 recognizes and binds to ubiquitinated cargo proteins. ESCRT-I and ESCRT-II then aid in cargo recruitment and the initiation of MVB membrane inward budding. The final step of ILV budding is performed by ESCRT-III, while VPS4 disassembles and recycles the ESCRT complexes for reuse [34,36,37,39,41,42]. The process is also assisted by accessory proteins like TSG101 and ALIX that regulate cargo selection and vesicle formation. In particular, the syndecan-syntenin-ALIX complex is a major player that helps coordinate these factors by ensuring their proper recruitment to MVBs at the right time and location [34,37,43,44,45,46]. In certain immune cells like dendritic cells, where the specificity of exosome cargo is highly critical for immune signaling, this ESCRT machinery is well-regulated [32,47,48,49,50,51]. As professional antigen-presenting cells, these cells utilize these pathways to package and release specific sets of membrane and cytosolic proteins, as well as nucleic acids, in exosomes [32,47,51]. In this way, they create very specific and functionally distinct exosomes that can travel around the body and tune the immune response by activating it, suppressing it, or inducing tolerance, depending on the state of the cell and the communication that it is trying to achieve [32,47,48,49,50,51]. In contrast, ESCRT-independent pathways offer alternative mechanisms for cargo sorting and ILV formation [34,39,52]. A key player in this alternative mechanism is ceramide, a lipid molecule that can induce a negative curvature in endosomal membranes, directly driving ILV budding, and thus determining the selective sorting of certain cargo, like proteolipid proteins [36,44,53]. Interestingly, more recent studies have also implicated ceramide-rich microdomains in a much more unconventional exosome biogenesis pathway that involves processes like nuclear envelope budding as well, suggesting a much wider scope for the mechanism in exosome formation [36,44,53,54]. Another contributor to ESCRT-independent biogenesis is a group of membrane proteins called tetraspanins. These proteins can organize specialized membrane domains and cluster specific cargoes to drive vesicle formation even without ubiquitination [34,39,45,52,55,56]. In addition, other lipid-driven processes like the production of phosphatidic acid by phospholipase D2 actively promote the recruitment of syntenin to drive exosome formation [36,57,58]. The final step of exosome release to the extracellular space is highly regulated by the Rab GTPases that mainly control MVB trafficking and docking at the plasma membrane, and SNARE proteins that mediate the membrane fusion events necessary for their secretion are the main orchestrators [59,60,61]. The sources of cargo that get selected for ILV incorporation are also extremely diverse: while ubiquitinated proteins are preferentially sorted through the ESCRT pathway, lipids and other RNA molecules can be incorporated via their interactions with RNA-binding proteins, or by direct recognition of specific sequence motifs [62,63]. In addition to these major players and mechanisms, there are other forms of regulation in place, like MVB acidification, precise calcium signaling, and even subtle changes in intracellular pH that all work together to fine-tune the efficiency and selectivity of the exosome biogenesis process [36,43,44]. In the context of cancer, exosome production is usually upregulated, and this is mainly driven by the phosphorylation of the various components of the syndecan-syntenin-ALIX complex, by aberrantly activated oncogenic signaling cascades [34,36,44]. In summary, a complex interplay of ESCRT complexes, lipid-driven mechanisms, accessory regulatory proteins, and more tightly controls exosome formation, cargo sorting and release, and subsequent secretion to allow exosomes to perform their diverse and often crucial functions in intercellular communication and overall physiological homeostasis.

## 3. Exosome Isolation and Characterization

Exosomes, nanosized vesicles secreted by nearly all cell types, have garnered significant attention as mediators of intercellular communication and potential biomarkers for various diseases. When studying exosomes, a crucial step is collecting and isolating them from various sources. This section covers the common pre-processing techniques and methods for isolation and characterization. Exosomes can be isolated from a wide variety of sources, including biofluids and cell culture supernatants [65]. Here is a more detailed look at common sources (Table 1):

Exosomes can be collected from the conditioned media of cultured cells. This allows researchers to study exosomes secreted by specific cell types in a controlled environment. As discussed earlier, many cell types from various biological kingdoms release exosomes, with the majority of research to date having focused on those derived from plants and animals [74]. The exosome yield can, however, vary significantly depending on the cellular source. For example, milk typically has a high exosome yield [75]. Thus, the choice of isolation method is critical for yield, purity, and processing costs and should be tailored to the specific source [75], as contaminating components vary. For example, blood samples can be contaminated with lipoproteins and albumin [67]. Some unconventional sources of exosomes, such as plants and fungi, are also being explored [67]. Traditional Methods for exosome isolation, while foundational, each has specific characteristics and limitations that influence their application in research. These methods are based on the physical properties of exosomes, such as size and density. Ultracentrifugation is a widely used method that involves multiple rounds of centrifugation at increasing speeds to separate particles based on size and density [67]. It is relatively low-cost and can be used for large volumes. However, it can result in low purity [76]. Density Gradient Centrifugation is often used after ultracentrifugation for further purification; this method separates exosomes based on their buoyant density [77]. The purest exosomes are obtained this way. Size exclusion chromatography separates molecules based on their size as they pass through a porous matrix [78]. Immunoaffinity Capture uses antibodies to specifically bind to exosomal surface proteins [78].

The major challenge in exosome research is the lack of a simple, reliable, and standardized isolation method [76]. Traditional techniques can be labor-intensive and may result in co-purification of other molecules [79]. To address these limitations, researchers have developed integrated biosensor-based methods for exosome separation and detection [76]. Some other considerations may include that the choice of isolation method can impact the yield, purity, and integrity of the isolated exosomes [77]. Some methods may require specialized equipment, such as ultracentrifuges [80]. In summary, while ultracentrifugation is often considered the “gold standard” [77], researchers are exploring new methods to improve exosome isolation and analysis [76]. Among the modern methods is microfluidics-based isolation, where microfluidic devices offer precise control over fluid flow, enabling efficient and high-throughput exosome isolation [79]. These devices can integrate various separation principles, such as size-based separation, which utilizes microchannels with specific dimensions to trap larger particles while allowing exosomes to pass through. Affinity-based separation incorporates antibodies or aptamers within the microchannels to selectively capture exosomes based on their surface markers. Acoustic Separation uses sound waves to manipulate and separate exosomes based on their size and density. Acoustic separation is label-free and can be integrated into microfluidic devices for continuous exosome isolation. The DEX are isolated from blood, and the lipoproteins co-purify because they have similar physicochemical properties. This is a big issue as the lipoproteins have been shown to directly interfere with the desired immunomodulatory function of DEX. A new report [81] documents this, and a new strategy to effectively deplete the lipoproteins. They show that this greatly increases the purity of the exosome preparation while maintaining the important exosomal markers. Exosome Precipitation involves using polymers to reduce the solubility of exosomes, causing them to aggregate and precipitate out of solution. While simple, it may co-precipitate other proteins. Lab-on-a-chip biosensing now makes it possible to integrate the isolation and characterization processes into a single platform [79]. These newer methods aim to improve upon the limitations of traditional techniques by offering enhanced purity, yield, and ease of use [76]. However, it is worth noting that exosome research still faces a lack of standardized isolation methods [76]. Table 2 below is the summary of different methods for exosome isolation—principle, procedure, advantages, and disadvantages.

Exosome characterization (Table 3) using advanced techniques such as nanoparticle tracking analysis, transmission electron microscopy, and dynamic light scattering can provide valuable insights into their size distribution, morphology, and concentration.

In a nutshell, when evaluating exosome isolation procedures, it is crucial to consider both yield and purity, as these parameters significantly influence downstream applications such as diagnostics, therapeutics, and molecular characterization. However, a major challenge in the field is the absence of standardized isolation protocols, which complicates the comparison of results across different laboratories. Variability in techniques, equipment settings, and sample types can lead to inconsistent outcomes, limiting reproducibility and hindering the development of universally accepted benchmarks. This lack of standardization not only affects inter-laboratory correlation but also raises concerns about the reliability and clinical applicability of exosome-based analyses [76].

## 4. Role of Exosomes in Cancer

**Figure 3:**
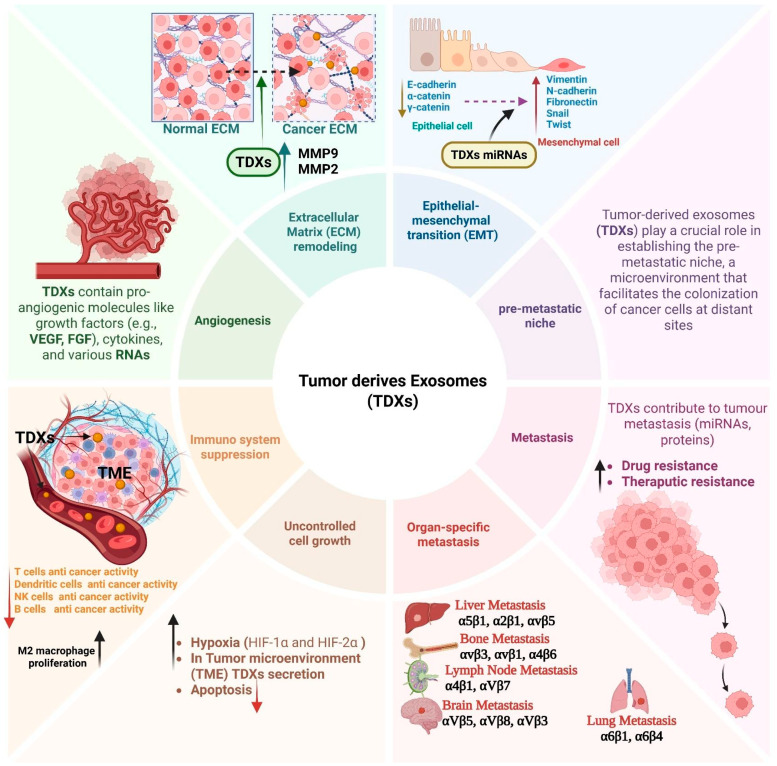
Tumor-derived exosomes (TDXs) role in cancer (created with biorender.com).

The tumor microenvironment (TME) is a highly complex, dynamic ecosystem. It is no longer perceived simply as a “bag of cancer cells” but is rather considered an intricate and active biological system consisting of a variety of supporting cells (stromal and immune), blood vessels, extracellular matrix, and a range of soluble factors [109,110,111,112]. Tumor growth and metastasis are also impacted by these heterogeneous cell–cell and cell–matrix networks. The most characteristic feature of TME, among various environmental stress factors, is hypoxia. Hypoxia is often caused by an imbalance between oxygen supply and consumption in TME [113,114,115,116,117]. The consumption of oxygen frequently exceeds the capacity for vascularization as a result of fast-growing tumors, leading to hypoxic (low oxygen) conditions in the tumor interstitium. When adapting to hypoxic stress, cancer cells utilize a transcription factor known as hypoxia-inducible factors (HIFs), which regulates various sets of genetic and metabolic changes [113,114,115,116,117]. HIFs enable the cells to adapt to low oxygen levels, which may support aggressive proliferation, as well as reprogram metabolism, and eventually cause the cancer cells to invade adjacent normal tissue to obtain resources for unlimited proliferation [116,118,119]. As tumor cells adapt to the resulting hypoxic conditions, the TME becomes more permissive to malignancy and metastasis [113,120]. Tumor cells in hypoxic conditions have a greater release of exosomes, nano vesicles that are important mediators of intercellular communication [121,122]. Tumor-derived exosomes (TDXs) regulate multiple stages of cancer development and progression (Figure 3). For instance, melanoma cells are particularly adept at efficiently releasing exosomes under hypoxic conditions, which significantly contributes to the disease’s aggressive nature [123,124,125]. TDXs are secreted more in hypoxic conditions and play a major role in remodeling the TME. TDXs contain a diverse range of cargoes, including proteins, lipids, and nucleic acids such as microRNAs, which can have a profound effect on the behavior of recipient cells [110,126,127]. A major role of TDXs is to promote angiogenesis, which is the formation of new blood vessels. This is crucial to provide the tumor with nutrients and oxygen. TDXs help in angiogenesis by delivering pro-angiogenic factors as well as exosomal microRNAs, including miR-210, which is enriched in exosomes derived from hypoxic tumor cells, to endothelial cells to stimulate their proliferation and migration [110,126,127,128,129]. This not only facilitates tumor growth and proliferation but also sets the stage for cancer cells to migrate through the bloodstream [128,129]. TDXs are also considered major players in reprogramming the immune landscape in the TME to suppress immune responses against the tumor. TDXs are able to reprogram immune cells within the TME to make the TME immunosuppressive [130,131,132]. For example, TEXs are able to reprogram macrophages in the TME from the antitumorigenic M1 state to the tumorigenic M2 state through exosomal miR-934, causing increased metastasis [133,134]. TDXs also promote the differentiation of monocytes into myeloid-derived suppressor cells (MDSCs), which suppress T cell responses through the secretion of various immunosuppressive factors such as PGE2 and TGF-β [29,135,136]. TDXs also inhibit T cell activation directly by carrying immunosuppressive molecules such as TGF-β and PD-L1, which inhibit T cell activation and promote regulatory T cell development [29,137,138,139]. Even the important antigen-presenting cells (APC), such as dendritic cells (DCs), can be tolerized by TDXs, resulting in a decreased ability of DCs to activate T cells, thus suppressing overall immune response [29,137,138,139]. Exosomes are also known to play a crucial role in metastasis [140]. They facilitate several critical processes involved in metastasis, such as epithelial to mesenchymal transition (EMT), which is a process by which epithelial cells lose their polarity and become mobile and invasive [140,141]. TDXs can deliver bioactive molecules that promote EMT, resulting in a significant increase in the metastatic potential of cancer cells [140,141]. For example, exosomal microRNA-106b-5p has been shown to promote colorectal cancer metastasis by modulating tumor-M2 macrophage crosstalk [142]. In addition, the surface proteins of exosomes, such as tetraspanins and integrins, have been shown to play important roles in organotropism and metastasis, and act as a homing signal that enables tumor cells in the circulation to home to specific organs and metastasize to those sites [56,143,144]. Resistance to therapy is one of the major roadblocks to cancer therapy. Recent evidence indicates that exosomes play a key role in this process by shuttling resistance genes and proteins between cells and, therefore, spreading resistance traits through the tumor [145,146,147]. For example, exosomes secreted from cisplatin-resistant lung cancer cells have been shown to transport miRNA-100-5p to drug-sensitive tumor cells, resulting in the activation of mTOR signaling and increased survival of drug-sensitive cells during chemotherapy [148,149]. In breast cancer, exosomes have also been shown to sequester HER2-targeted drugs or even reprogram the gene expression of recipient tumor cells to decrease the effectiveness of anti-cancer drugs [150,151]. In conclusion, exosomes are not merely waste products of tumor growth but are active and versatile participants in almost every aspect of cancer biology [152,153], from supporting angiogenesis and immune evasion to facilitating metastasis and contributing to therapeutic resistance. Their diverse roles make them not just a significant challenge in cancer treatment but also a promising target for therapeutic intervention [152,154]. Ongoing research into the various roles of exosomes in the TME is not only broadening our understanding of cancer progression but also opening up new avenues for the development of effective and targeted therapies.

## 5. DC-Derived Exosomes in Therapeutic Application

Dendritic cell-derived exosomes (DEXs) are multifunctional nanocarriers with considerable promise in cancer immunotherapy [155,156,157]. Mature DEXs exhibit critical immune molecules like MHC-1 and MHC-II, which are essential for tumor antigen presentation to T cells. Apart from MHC molecules, the activation of antigen-specific cytotoxic T lymphocytes requires co-stimulatory proteins such as CD80 and CD86, which are carried abundantly by DEXs [158]. In comparison to whole dendritic cell therapy, DEXs perform better as candidates in cancer immunotherapy since they can resist the suppression in the tumor microenvironment (TME) [159]. Moreover, their application is widely being explored because reports have shown that exosomes secreted by dendritic cells have been regarded highly efficacious since they have been shown to possess good storage stability, safety with high biocompatibility, and content [160,161]. Dendritic cell-derived exosomes can also be engineered to express immunostimulatory molecules, antigens, or fusion proteins that enhance their therapeutic potential [6]. Exosome-mediated drug delivery is an innovative technology that presents distinct advantages compared to conventional drug delivery systems. One of the most commonly known advantages of using exosome-based drug delivery, like DEXs, is the fact that dendritic cells naturally originate from the human body, meaning that it is highly biocompatible with low immunogenicity [162]. DEX-based drug delivery also has a leverage over its proven barrier crossing [163]. Multiple studies have elucidated DEX’s capability to travel through difficult biological barriers like the blood–brain barrier (BBB), which is known for its highly selective permeability from the bloodstream to the brain. Although its layers protect the brain from infections or toxins, it is also challenging to deliver drugs. DEXs are advantageous in the context of having recognizable molecules that allow them to pass and deliver their cargo. This characteristic makes them highly valuable in targeting malignancies beyond the brain [164]. Dendritic cell-derived exosomes rely on accomplished mechanisms, techniques, and strategies to fully leverage their therapeutic potential. Passive and active methods of drug loading are the two fundamental types that influence their release profiles, loading efficiency, and the distribution of the candidate drug. Incubation is a passive loading approach that involves mixing the DEXs with the drug and leaving it at an appropriate temperature to permit the drug to be infused or adsorbed into the bilayer of the exosome [165]. Sonication is an active loading method that requires temporary disruption of the exosome’s membrane for drug entry. This can be achieved by exposing DEXs to ultrasonic waves with appropriate optimization via a sonicator [166]. One of the first utilizations of DEXs in cancer immunotherapy was to treat non-small cell lung cancer (NSCLC) [167]. Patients from phase I of the study received DEX-loaded MAGE tumor antigens. These peptides are small pieces of tumor antigens that stimulate CD8+ T cells, often known as cytotoxic T lymphocytes [168]. The study reported that there is an increase in NK cell activity, which is an indication of the tumor cells being exterminated by the patient’s NK cells. The clinical investigation by Morse et al. 2005 was the first to demonstrate DEXs as a cell-free cancer immunotherapy in solid tumors and eventually became the foundation study in paving the way for more future advancements in the field [167]. Apart from NSCLC, DEXs were also used to treat hepatocellular carcinoma (HCC), a malignancy in the kidney. This time, DEXs were bioengineered to carry alpha-fetoprotein (AFP) as their cargo [169]. DEXs-based clinical trial phase I and II indicated that limitations promote anti-cancer activity [170]. Mature dendritic cell-derived exosomes are promising immune therapeutic tools for cancer [171]. In solid tumors, mature DEXs prime tumor antigen and activate Th1 and CD8+ T cells, which show a promising outcome against cancer [172]. DEXs also work as a potential immune response against cancer cells, and this is an effective cell-free immunotherapeutic approach [173]. Compared to the DC vaccine approach, DEXs are more effective due to their MHCI and MHCII capabilities, which promote a strong immunome response [174]. Research evidence suggests that engineered DEX shows promising outcomes in an in vivo breast cancer model [175].

**Figure 4:**
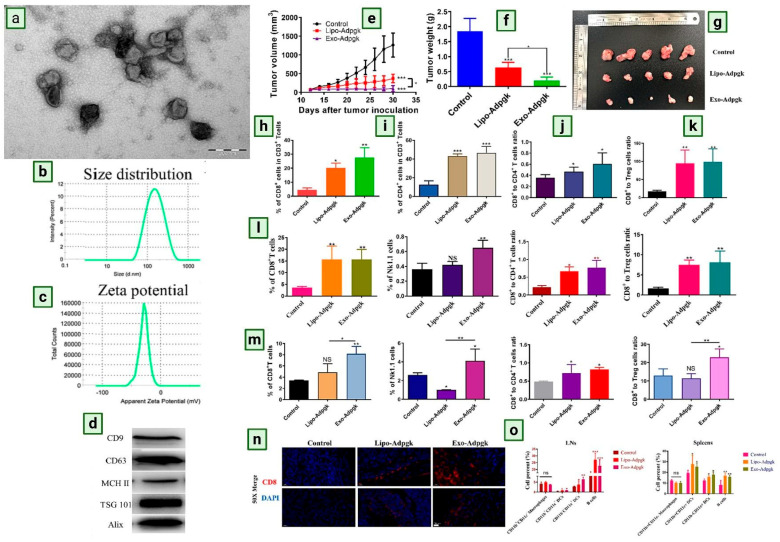
Dendritic cell-derived exosomes-based anti-cancer application. Dendritic cell-derived exosomes TEM images (**a**), size (**b**), and zeta potential (**c**) of exosomes measured by DLS. Scale bar: 200 nm. Maker proteins on exosomes by Western blot analysis (**d**). Antitumor response of Exo-Adpgk on MC-38 tumors of C57BL/6 mice. Tumor volume–time curve (**e**), tumor weight (**f**), and pictures of the tumors (**g**) isolated from mice on day 30 after the MC-38 inoculation. Proportions of CD8^+^ T cells (**h**), CD4^+^ T cells (**i**), CD8^+^ CTL to CD4^+^ ratios (**j**), and CD8^+^ CTL to Treg ratios (**k**). The frequency of CD8^+^ T cells, NK1.1 cells as well as CD8^+^ T/CD4^+^ T, CD8^+^ T/Treg ratios in LNs (**l**) and spleens (**m**) from mice of various treatments. (**n**) Representative immunofluorescence of CD8^+^ T cells (red) in B16F10 melanoma tumors (blue). Tumors’ cryostat sections were stained with CD8 antibody (red), and tumor nuclei were stained with DAPI (blue). (**o**) Quantified APCs (including CD11b^+^CD11c^−^ Macrophage, CD11b^+^CD11c^+^ DCs, CD11b^+^CD11c^−^ DCs, and B cells) populations in LNs and spleens from vaccinated mice by flow cytometry analysis. (Reproduced with permission under Creative Commons CC BY-NC-ND 4.0 license from ref. [13] Copyright 2023, Elsevier publisher).

Research evidence for DEX-based cancer therapeutic applications is depicted in Figure 4.

Since AFP is abnormally overexpressed in adults with HCC, it is adopted as a biomarker for assessing the prognosis of liver cancer patients [176]. However, the immune responses fail to recognize AFP as foreign, as it is an antigen that is produced during the development of the fetus, which leads to immune tolerance during adulthood [176]. The immune system is also actively being suppressed by HCC by creating an immunosuppressive tumor microenvironment (TME) through the upregulation of immune checkpoints like PD-L1, efficiently impeding T-cell functions [176,177]. These challenges were solved with AFP-loaded DEXs, genetically modified to present AFP in a more immunogenic way to re-activate the host’s immune system [169,178]. By presenting AFP on MHC molecules coupled with necessary co-stimulatory signals, DEXs promote T-cell activation, break immune tolerance, and change the tumor microenvironment towards facilitating anti-tumor immunity. Therefore, DEX-based delivery has emerged as a promising approach to stimulate effective antigen-specific immune responses in HCC [179].

## 6. Clinical Trial of Dendritic Cell-Derived Exosomes

The clinical trial ID NCT01159288 (Table 4) examines a cell-free immunotherapy approach, where tumor antigen-loaded DEX is applied against non-small lung cancer (NSCLC). In this trial, DEXs are isolated from cancer patients. Gustave Roussy and Curie institutes have developed an immunotherapy involving metronomic cyclophosphamide (mCTX) followed by vaccinations with tumor antigen-loaded DEXs. mCTX inhibits Treg functions, restoring T and NK cell effector functions, and DEXs are able to activate the innate and adaptive immunity. Phase I trials showed the safety and feasibility of DEX vaccines, but no induction of T cells could be monitored in patients. Since 2007, a novel process has been developed and validated for the isolation of second-generation DEX, which demonstrated improved immunostimulatory capacities. A clinical trial was subsequently proposed to evaluate a maintenance immunotherapy regimen using this DEX-based treatment in 47 patients with advanced, unresectable non-small cell lung cancer (NSCLC). The primary objective of the study was to enhance the progression-free survival (PFS) rate at four months in patients who had shown a positive response or disease stabilization following induction chemotherapy. A phase I clinical trial of autologous exosomes in melanoma patients established the safety and large-scale manufacturing feasibility, with a partial clinical response in a single patient and stable disease in others, despite an absence of detectable peripheral MAGE3-specific T cell responses [174,180]. In addition to safety and manufacturability, a major obstacle to the advancement of DEX into Phase III trials has been its low immunogenicity and the technical challenge of eliciting robust, durable T-cell responses [174,180].

## 7. Challenges and Future Prospects

**Figure 5:**
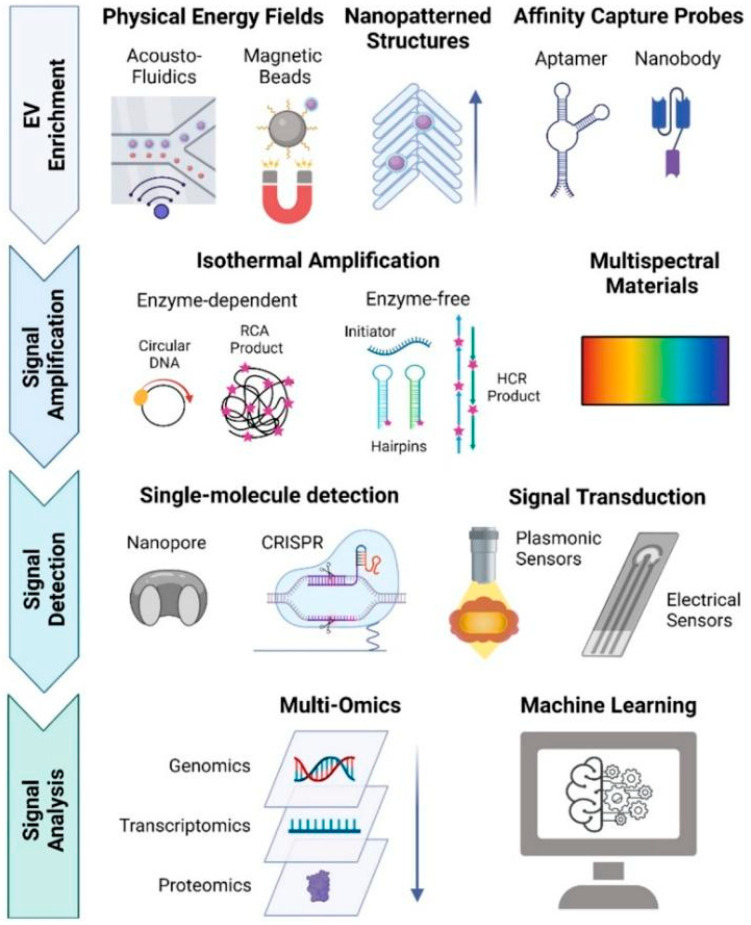
Single exosome profiling approach. (Adapted with permission from ref. [181] Copyright @ 2022 American Chemical Society.)

Exosomes research effectively transforms cancer theranostics research. There are still some domains that are developing support for the progression of exosome research. We need to develop a standard isolation procedure with high yield, high purity, and reproducibility. Exosome heterogeneity is one of the complications in exosome research. Multiple factors regulate it, such as exosome origin, size, and molecular diversity [181]. The single-exosome profiling approach (Figure 5) addresses this complexity, thereby facilitating the development of exosome-based biomarkers and advancing precision cancer therapeutics [181]. The multi-omics approach plays a significant role in revealing exosomes’ molecular signature. Such high-throughput methods can reveal a global perspective of DEX heterogeneity and define specific subsets, and predict the functional outcome of each subset. This detailed information is critical for defining adequate quality control measures and avoiding the development of unpredictable off-target effects, such as immune activation/autoimmunity, for safe and predictable DEX therapies [106]. Large-scale production is a big challenge for exosome-based translational research and clinical trials. Exosome-based drug delivery overcomes several traditional issues in drug delivery, but in the case of the exosome loading approach, it is still under standardization [182]. Regulation and safety concern frameworks based on exosome-based theranostics are still evolving. Ensuring safety, minimizing immunogenicity, and establishing standard quality control measures are essential before clinical approval. DEX is not able to activate effective T cell-mediated anti-cancer activity; dendritic cell-like DEX cannot migrate to the tumor location; and heterogeneity and standardization challenge, large-scale production and storage, and several clinical trials indicate limited efficiency [183,184,185]. The development of advanced nanotechnologies for exosome isolation, such as microfluidic and affinity column-based techniques, has enabled the production of high-yield and high-purity preparations [186,187]. DEX-based cancer therapeutics’ major limitation is that this approach does not induce sufficient T-mediated anti-cancer immune response [188]. Current research evidence reports that after modifications, DEX has become a promising therapeutic tool for cancer [189]. Exosome-based cancer translational research takes more time for proper clinical trial-based validation. Finally, after overcoming all challenges, exosomes may open a cutting-edge personalized medicine era for cancer.

## 8. Conclusions

DEXs have emerged as a next-generation platform in cancer immunotherapy, offering a unique, cell-free approach that harnesses the immune-stimulating properties of dendritic cells while addressing the maximum limitations and challenges of conventional DC-based vaccines. With their inherent capacity to present tumor-associated antigens (TAAs), activate T lymphocytes, and modulate the immune microenvironment, DEXs have demonstrated considerable potential to initiate and amplify a robust anti-tumor immune response in both preclinical studies and early-phase clinical trials. Their nano-sized morphology, stability, high biocompatibility, low immunogenicity, and potential for systemic delivery of bio-active molecular cargoes make them an attractive therapeutic tool in the growing field of cancer nanomedicine and personalized immunotherapy. Despite their significant potential, several challenges still limit the clinical translation of DEX-based therapies. One of the major hurdles is the heterogeneity of exosomes themselves, not only among different DC subsets but also due to variations introduced during ex vivo culture and exosome isolation. This heterogeneity impacts therapeutic predictability, targeting specificity, and immune potency. Another major limitation is the lack of standardized, scalable, and reproducible isolation and purification methods. Ultracentrifugation, size-exclusion chromatography, and polymer-based precipitation often yield heterogeneous populations with variable purity, which may affect immunogenic outcomes and make it difficult to obtain consistent results each time. In addition, antigen-loading strategies remain suboptimal, with limited efficiency in incorporating specific tumor antigens or immune modulators into exosomes without compromising vesicle integrity. On top of that, the pharmacokinetics, bio-distribution, and understanding of the mechanisms of DC-derived exosomes in vivo are not yet fully transparent, which complicates dose optimization and regulatory assessment in the clinical translation stage. Moreover, from an immunological perspective, the tumor microenvironment (TME) constitutes a major barrier by limiting the recruitment and functional activity of exosome-activated immune cells. Tumor-associated immunosuppression, such as the presence of regulatory T cells (Tregs), myeloid-derived suppressor cells (MDSCs), and inhibitory cytokines, can blunt DEX-mediated responses. Overcoming these barriers will require synergistic strategies that incorporate DEXs modification with immune checkpoint inhibitors, adjuvants, or TME-modulating agents to design personalized cancer immunotherapy and move one step closer to precision oncology. Looking toward the future outlook, several perspectives are likely to shape the evolution of DC-derived exosome-based cancer immunotherapy. Advances in exosome engineering, including surface modification with targeting ligands, application of click chemistry, membrane fusion techniques, and synthetic biology approaches, may significantly improve antigen loading and immune activation profiles. Integration of CRISPR-based gene editing and nanotechnology could allow for the development of smart, programmable exosomes tailored for specific tumor types or patient immunoprofiles (personalized immunotherapy). Furthermore, multi-omics approaches, including proteomics, transcriptomics, and lipidomics, combined with artificial intelligence (AI) and advanced machine learning (ML) algorithms-based analytic tools, are expected to enhance exosome characterization and predict personalized therapeutic responses with higher precision and accuracy. Most importantly, efforts must also focus on developing Good Manufacturing Practice (GMP) and compliant protocols to ensure large-scale production, quality control, and regulatory acceptance of exosome-based therapies. In conclusion, dendritic cell-derived exosomes represent a promising and adaptable platform for cancer immunotherapy. Despite the existing challenges in scalability, targeting, and regulation, ongoing research and innovation could enable their successful clinical translation in the future. With continued support and collaboration, DEX may soon rise as a beacon of hope, reshaping the landscape of precision immune-oncology with sophistication and precision.

## Figures and Tables

**Table 1:** Recent clinical advances of exosome-based theranostics.

Exosome for Liquid Biopsy for Cancer		
Exosome Source	Clinical Significance	References
Blood (Plasma/Serum)	Blood is a complex biofluid containing exosomes from various cell types. Plasma and serum are commonly used for exosome isolation due to their accessibility. However, they also contain abundant proteins like albumin and lipoproteins that can contaminate exosome preparations.	[65,66,67]
Urine	Exosomes are present in urine and can be valuable for studying kidney function and urological diseases.	[65]
Saliva	Saliva offers a non-invasive source of exosomes, useful for oral and systemic disease diagnostics.	[66]
Breast Milk	Breast milk is rich in exosomes, which play a role in infant immunity and development.	[65]
Amniotic Fluid	Exosomes in amniotic fluid can provide insights into fetal development and pregnancy-related complications.	[65]
Cerebrospinal Fluid	CSF-derived exosomes are valuable for studying neurological disorders.	[65]
Ascites Fluid	This fluid, found in the abdominal cavity of patients with certain cancers, contains exosomes that can provide information about the tumor microenvironment.	[65]
Sweat	Biomarker source	[68]
Tear	Biomarker source	[69]
**Exosomes sources for therapeutic development**		
**Exosome source**	**Clinical significance**	**References**
Plant cell	Use as a therapeutic tool	[70]
Immune cell	Use as a therapeutic tool	[71]
Stem cell	Use as a therapeutic tool	[72]
Tumor cell	Use as a therapeutic tool (not recommended due to its enrichment of oncogenic cargo)	[73]

**Table 2:** Comparison of different exosomes isolation methods.

Methods	Principle	Procedure	Advantages	Disadvantages	References
Differential Ultracentrifugation	This method involves multiple rounds of centrifugation at progressively higher speeds. Each step pellets particles of a certain size and density, allowing for the enrichment of exosomes.	The sample is first centrifuged at low speeds (e.g., 300× *g*, then 2000× *g*) to remove cells, cell debris, and larger vesicles. The supernatant is then centrifuged at a higher speed (e.g., 10,000× *g*) to separate exosomes from larger, non-exosomal vesicles. Finally, the supernatant is ultracentrifuged at very high speed (e.g., 100,000–150,000× *g*) to pellet the exosomes. The exosome pellet can be resuspended in a suitable buffer for downstream analysis	Can be used for large volumes, relatively low cost, and with no need for additional chemical reagents	Can be time-consuming and may result in low purity due to the co-purification of other molecules. Exosomes might be damaged during the process	[67,82,83]
Density Gradient Ultracentrifugation	This method separates particles based on their buoyant density. The sample is layered on top of a density gradient medium (e.g., sucrose or iodixanol) and centrifuged until particles reach their equilibrium density.	After differential ultracentrifugation, the exosome-containing pellet is resuspended and placed on top of a density gradient Ultracentrifugation is performed until the exosomes migrate to their corresponding density within the gradient. Fractions are collected from the gradient, and exosome-containing fractions are identified and pooled	Higher purity compared to differential ultracentrifugation	More complex and time-consuming than differential ultracentrifugation, as well as low yields	[77,84,85]
Size Exclusion Chromatography (SEC)	SEC separates molecules based on their size as they pass through a porous matrix. A column is packed with a stationary phase consisting of porous beads. Smaller molecules enter the pores and take a longer, more tortuous path, eluting later. Larger particles, like exosomes, cannot enter the pores and elute earlier.	The sample is loaded onto the SEC column. A buffer is used to elute the sample through the column. Fractions are collected as they elute from the column. Exosome-containing fractions are identified based on their elution volume.	SEC can separate exosomes based on their size Gentle method that preserves exosome integrity	A limited quantity of EVs recovered.	[78,86,87]
Filtration	Filtration methods use filters with defined pore sizes to separate particles based on size. Ultrafiltration membranes with specific molecular weight cut-offs are commonly used to enrich exosomes	The sample is passed through a filter membrane with a specific pore size. Particles larger than the pore size (including exosomes) are retained on the filter Smaller molecules pass through the filter. The retained exosomes can be recovered by back-flushing the filter or dissolving them in a suitable buffer.	Relatively simple and rapid. Can be used to concentrate exosome samples	Membrane clogging can be an issue. Exosomes may be damaged by shear forces during filtration.	[77,80,88]
Precipitation with Polymers	This method involves using polymers to reduce the solubility of exosomes in solution. The polymers bind to water molecules, effectively forcing exosomes to aggregate and precipitate out of the solution	The polymer solution is added to the sample containing exosomes. The mixture is incubated for a specific time and temperature to allow for exosome precipitation. The precipitated exosomes are then collected by centrifugation. The exosome pellet is resuspended in a suitable buffer for downstream analysis.	Simple and relatively inexpensive. Does not require specialized equipment like ultracentrifuges	Co-precipitation of other non-exosomal contaminants, such as proteins and polymeric materials, is unavoidable. May be less pure compared to other methods like ultracentrifugation or size exclusion chromatography. The choice of polymer and precipitation conditions can affect exosome yield and purity.	[89,90,91]
Immunoaffinity Capture	This method uses the specific binding between an antibody and an exosomal surface protein to selectively capture exosomes. Antibodies against specific exosomal markers (e.g., CD9, CD63, CD81) are immobilized on a solid support (e.g., beads, columns, or microplates). When a sample containing exosomes is incubated with the antibody-conjugated support, exosomes expressing the target protein are captured.	Antibodies against exosomal surface markers are immobilized on a solid support. The sample containing exosomes is incubated with the antibody-conjugated support, allowing the exosomes to bind to the antibodies. The solid support is washed to remove unbound material. The captured exosomes are then eluted from the support, typically by changing the pH or ionic strength of the buffer	High specificity for exosomes expressing the target protein. Can be used to isolate specific subpopulations of exosomes.	Requires knowledge of exosomal surface markers. Antibody availability and cost can be limiting factors Elution steps can result in sample loss, making the method less suitable for downstream analysis.	[78,91,92].
Microfluidic	Microfluidic exosome separation leverages the unique physical and chemical properties of exosomes in a controlled microenvironment. These properties include size, surface markers, deformability, and electrical characteristics.	Size-based separation: This method separates exosomes based on their size using microchannels with precisely controlled dimensions. Dynamic methodologies: Separation based on other properties, such as electrical characteristics.	Enhanced purity: Microfluidic systems can achieve higher purity compared to ultracentrifugation. Cost-effectiveness: Microfluidic technologies offer a cost-effective solution for exosome isolation.	Technological immaturity: Exosome research using microfluidics is still in its early stages. Lack of standardization: The absence of standardized methods for exosome separation can lead to suboptimal inter-laboratory correlation and difficulty in comparing studies. Challenges in isolating exosomes: The inherent heterogeneity of exosomes and the complexity of biofluids pose significant challenges for their isolation.	[93,94,95]

**Table 3:** Different exosome characterization techniques.

Characterization Types	Importance	Reference
Physical Characterization		
Nanoparticle Tracking Analysis	NTA is utilized for concentration measurements and size distribution curves of exosomes. NTA operates by tracking the Brownian motion of individual particles in a sample using light scattering. By measuring the rate of movement, the software calculates the hydrodynamic diameter of each particle using the Stokes-Einstein equation.	[96,97,98]
Dynamic Light Scattering	DLS, as well as NTA, fluorescence signals, and flow cytometry, are optical methods used to characterize vesicles.	[99]
Electron Microscopy (TEM/SEM):	Traditional detection techniques utilized to quantify the isolated exosomes include scanning electron microscopes and transmission electron microscopes.	[98]
NanoFCM	Nano-flow cytometry is a quantitative and qualitative measurement of single EVs, like exosomes (it is applicable for cell culture suspension and body fluid).	[100]
Super-Resolution Microscopy (SRM)	SRM works with Oxford Nano Imaging (ONI) and supports the decoding of exosomes’ morphology, tracking of EV uptake, cargo composition, and heterogeneity.	[101,102]
**Characterization of Molecules**		
Western Blotting	Western blotting can be performed using exosomal markers such as CD9, CD63, and CD81.	[98]
Flow Cytometry	It is one of the optical methods for characterizing vesicles. It can identify and characterize the cytoplasmic or surface proteins of EVs.	[99]
Exoview Chip	This is a microarray-based method where affinity-based antibodies are captured for exosomes’ surface markers, such as CD9, CD63, and CD81. This capture visualization by the supporter of ExoViewR100	[103,104]
Analysis of Proteomes and Genomes	Mass Spectrometry, RNA-seq: These techniques are used for in-depth analysis of exosome contents.	[105,106]
Quantification and Evaluation of Purity	Quantifying protein (BCA/Bradford test): These are standard methods for determining the overall protein concentration in the exosome sample. Finding contaminants (how well they remove albumin and lipoprotein): It is important to assess the presence of contaminating proteins like albumin and lipoproteins to determine the effectiveness of the isolation method.	[107]
Functional Analysis	Studies on uptake (cell interaction tests and fluorescent labeling): These assays help to understand how exosomes interact with and are taken up by target cells. Functional assays in vitro and in vivo: These assays assess the biological effects of exosomes on cells or in living organisms.	[108]

**Table 4:** Clinical trial of dendritic cell-derived exosomes.

Clinical Trials ID	Status	Cancer Types	Clinical Significant	Sponsor
NCT01159288	Completed	advanced non-small cell lung cancer (NSCLC)	Trial of a Vaccination With Tumor Antigen-loaded Dendritic Cell-derived Exosomes (CSET 1437)	Gustave Roussy, Cancer Campus, Grand Paris
Key insights from the clinical trial: **Phase:** Phase II **Objective:** To assess the efficacy of IFN-γ-DEXs as maintenance immunotherapy after platinum-based chemotherapy in advanced NSCLC. **Patient Groups** **Number Enrolled/Treated:** 26 enrolled/22 treated **Diagnosis:** Advanced (stage IIIB/IV) unresectable NSCLC **Key Criteria:** HLA-A2 positive, no progression after 4 cycles of platinum-based chemotherapy. **Baseline:** 64% had adenocarcinoma, 82% had stage IV disease. **Interventions** **Treatment:** IFN-γ-matured dendritic cell-derived exosomes (IFN-γ-DEXs) loaded with MAGE tumor antigens. Dosing: 0.13 μg MHC class II molecules per injection, administered intravenously. **Schedule:** Weekly for the first 4 vaccinations, followed by boosters administered every 2 weeks for 3 boosters, then monthly boosters. **Adjunct Therapy:** Oral metronomic cyclophosphamide (50 mg/day) to inhibit T-regs. **Endpoint** **Primary:** Progression-free survival (PFS) rate at 4 months. **Secondary:** Overall survival (OS), time to progression (TTP), safety, and immunological response. **Key Findings** **Efficacy** (Primary Endpoint)**:** Not met. Only 32% (7/22) of patients were progression-free at 4 months (target was >50%). Median PFS was 2.2 months. **Overall Survival (OS):** Median OS was 15 months. **Safety:** Treatment was well-tolerated. One patient experienced a Grade 3 dose-limiting hepatotoxicity. **Immunological Response:** No MAGE-specific CD8+ T-cell responses were detected. An increase in NKp30-dependent Natural Killer (NK) cell function was observed and was correlated with longer PFS. Clinical benefit was associated with higher levels of the NK ligand BAG6 on the exosomes.				

Source: https://clinicaltrials.gov/. (accessed on 17 September 2025)

## Data Availability

Data sharing is not applicable to this article as no datasets were generated or analyzed during the current study.

## References

[1] Guida F., Kidman R., Ferlay J., Schüz J., Soerjomataram I., Kithaka B., Ginsburg O., Vega R.B.M., Galukande M., Parham G. (2022). Global and regional estimates of orphans attributed to maternal cancer mortality in 2020. Nat. Med..

[2] Zafar A., Khatoon S., Khan M.J., Abu J., Naeem A. (2025). Advancements and limitations in traditional anti-cancer therapies: A comprehensive review of surgery, chemotherapy, radiation therapy, and hormonal therapy. Discov. Oncol..

[3] Ghemrawi R., Abuamer L., Kremesh S., Hussien G., Ahmed R., Mousa W., Khoder G., Khair M. (2024). Revolutionizing Cancer Treatment: Recent Advances in Immunotherapy. Biomedicines.

[4] Theivendren P., Kunjiappan S., Pavadai P., Ravi K., Murugavel A., Dayalan A., Kumar A.S.K. (2024). Revolutionizing Cancer Immunotherapy: Emerging Nanotechnology-Driven Drug Delivery Systems for Enhanced Therapeutic Efficacy. ACS Meas. Sci. Au.

[5] Santos P., Almeida F. (2021). Exosome-Based Vaccines: History, Current State, and Clinical Trials. Front. Immunol..

[6] Sonar S., Das A., Kalele K., Subramaniyan V. (2025). Exosome-based cancer vaccine: A cell-free approach. Mol. Biol. Rep..

[7] Mehta N.K., Moynihan K.D., Irvine D.J. (2015). Engineering New Approaches to Cancer Vaccines. Cancer Immunol. Res..

[8] Wang H., Najibi A.J., Sobral M.C., Seo B.R., Lee J.Y., Wu D., Li A.W., Verbeke C.S., Mooney D.J. (2020). Biomaterial-based scaffold for in situ chemo-immunotherapy to treat poorly immunogenic tumors. Nat. Commun..

[9] Zhou Y., Wei Y., Tian X., Wei X. (2025). Cancer vaccines: Current status and future directions. J. Hematol. Oncol..

[10] Kaur P., Mehrotra R., Rengaswamy S., Kaur T., Hariprasad R., Mehendale S.M., Rajaraman P., Rath G.K., Bhatla N., Krishnan S. (2017). Human papillomavirus vaccine for cancer cervix prevention: Rationale & recommendations for implementation in India. Indian J. Med. Res..

[11] Lee K.-W., Yam J.W.P., Mao X. (2023). Dendritic Cell Vaccines: A Shift from Conventional Approach to New Generations. Cells.

[12] Yao Y., Fu C., Zhou L., Mi Q.-S., Jiang A. (2021). DC-Derived Exosomes for Cancer Immunotherapy. Cancers.

[13] Li J., Li J., Peng Y., Du Y., Yang Z., Qi X. (2022). Dendritic cell derived exosomes loaded neoantigens for personalized cancer immunotherapies. J. Control. Release.

[14] Lee Y.J., Shin K.J., Chae Y.C. (2024). Regulation of cargo selection in exosome biogenesis and its biomedical applications in cancer. Exp. Mol. Med..

[15] Tenchov R., Sasso J.M., Wang X., Liaw W.-S., Chen C.-A., Zhou Q.A. (2022). Exosomes─Nature’s Lipid Nanoparticles, a Rising Star in Drug Delivery and Diagnostics. ACS Nano.

[16] Li Y., Chen Z.-K., Duan X., Zhang H.-J., Xiao B.-L., Wang K.-M., Chen G. (2022). Targeted inhibition of tumor-derived exosomes as a novel therapeutic option for cancer. Exp. Mol. Med..

[17] Sohrabi B., Dayeri B., Zahedi E., Khoshbakht S., Pour N.N., Ranjbar H., Nejad A.D., Noureddini M., Alani B. (2022). Mesenchymal stem cell (MSC)-derived exosomes as novel vehicles for delivery of miRNAs in cancer therapy. Cancer Gene Ther..

[18] Jung I., Shin S., Baek M.-C., Yea K. (2024). Modification of immune cell-derived exosomes for enhanced cancer immunotherapy: Current advances and therapeutic applications. Exp. Mol. Med..

[19] Sonar S., Anand K. (2024). Plant-derived exosomes: A Green Nanomedicine for Cancer. Clin. Transl. Discov..

[20] Das A., Sonar S., Kalele K., Subramaniyan V. (2024). Milk exosomes: Harnessing nature’s duality for cancer therapy. Clin. Transl. Discov..

[21] Pitt J.M., André F., Amigorena S., Soria J.-C., Eggermont A., Kroemer G., Zitvogel L. (2016). Dendritic cell–derived exosomes for cancer therapy. J. Clin. Investig..

[22] Nikfarjam S., Rezaie J., Kashanchi F., Jafari R. (2020). Dexosomes as a cell-free vaccine for cancer immunotherapy. J. Exp. Clin. Cancer Res..

[23] Wang S., Shi Y. (2022). Exosomes Derived from Immune Cells: The New Role of Tumor Immune Microenvironment and Tumor Therapy. Int. J. Nanomed..

[24] Meng Y., Yao Z., Ke X., Hu M., Ren H., Gao S., Zhang H. (2024). Extracellular vesicles-based vaccines: Emerging immunotherapies against cancer. J. Control. Release.

[25] Besse B., Charrier M., Lapierre V., Dansin E., Lantz O., Planchard D., Le Chevalier T., Livartoski A., Barlesi F., Laplanche A. (2016). Dendritic cell-derived exosomes as maintenance immunotherapy after first line chemotherapy in NSCLC. OncoImmunology.

[26] Blumenschein G.R., Devarakonda S., Johnson M., Moreno V., Gainor J., Edelman M.J., Heymach J.V., Govindan R., Bachier C., de Spéville B.D. (2022). Phase I clinical trial evaluating the safety and efficacy of ADP-A2M10 SPEAR T cells in patients with MAGE-A10^+^ advanced non-small cell lung cancer. J. Immunother. Cancer.

[27] Tian H., Li W. (2017). Dendritic cell-derived exosomes for cancer immunotherapy: Hope and challenges. Ann. Transl. Med..

[28] Xia J., Miao Y., Wang X., Huang X., Dai J. (2022). Recent progress of dendritic cell-derived exosomes (Dex) as an anti-cancer nanovaccine. Biomed. Pharmacother..

[29] Hosseini R., Asef-Kabiri L., Yousefi H., Sarvnaz H., Salehi M., Akbari M.E., Eskandari N. (2021). The roles of tumor-derived exosomes in altered differentiation, maturation and function of dendritic cells. Mol. Cancer.

[30] Zhang F., Guo J., Zhang Z., Qian Y., Wang G., Duan M., Zhao H., Yang Z., Jiang X. (2022). Mesenchymal stem cell-derived exosome: A tumor regulator and carrier for targeted tumor therapy. Cancer Lett..

[31] Jin X., Zhang J., Zhang Y., He J., Wang M., Hei Y., Guo S., Xu X., Liu Y. (2024). Different origin-derived exosomes and their clinical advantages in cancer therapy. Front. Immunol..

[32] Redkin T., Turubanova V. (2025). Dendritic cell-derived exosomes as anti-cancer cell-free agents: New insights into enhancing immunogenic effects. Front. Immunol..

[33] Hussen B.M., Faraj G.S.H., Rasul M.F., Hidayat H.J., Salihi A., Baniahmad A., Taheri M., Ghafouri-Frad S. (2022). Strategies to overcome the main challenges of the use of exosomes as drug carrier for cancer therapy. Cancer Cell Int..

[34] Krylova S.V., Feng D. (2023). The Machinery of Exosomes: Biogenesis, Release, and Uptake. Int. J. Mol. Sci..

[35] Hessvik N.P., Llorente A. (2018). Current knowledge on exosome biogenesis and release. Cell. Mol. Life Sci..

[36] Han Q.-F., Li W.-J., Hu K.-S., Gao J., Zhai W.-L., Yang J.-H., Zhang S.-J. (2022). Exosome biogenesis: Machinery, regulation, and therapeutic implications in cancer. Mol. Cancer.

[37] Woodman P.G., E Futter C. (2008). Multivesicular bodies: Co-ordinated progression to maturity. Curr. Opin. Cell Biol..

[38] Rädler J., Gupta D., Zickler A., EL Andaloussi S. (2023). Exploiting the biogenesis of extracellular vesicles for bioengineering and therapeutic cargo loading. Mol. Ther..

[39] Tschuschke M., Kocherova I., Bryja A., Mozdziak P., Volponi A.A., Janowicz K., Sibiak R., Piotrowska-Kempisty H., Iżycki D., Bukowska D. (2020). Inclusion Biogenesis, Methods of Isolation and Clinical Application of Human Cellular Exosomes. J. Clin. Med..

[40] Mageswaran S.K., Dixon M.G., Curtiss M., Keener J.P., Babst M. (2013). Binding to Any ESCRT Can Mediate Ubiquitin-Independent Cargo Sorting. Traffic.

[41] Shim S., Merrill S.A., Hanson P.I. (2008). Novel Interactions of ESCRT-III with LIP5 and VPS4 and their Implications for ESCRT-III Disassembly. Mol. Biol. Cell.

[42] Christ L., Raiborg C., Wenzel E.M., Campsteijn C., Stenmark H. (2017). Cellular Functions and Molecular Mechanisms of the ESCRT Membrane-Scission Machinery. Trends Biochem. Sci..

[43] McAndrews K.M., Kalluri R. (2019). Mechanisms associated with biogenesis of exosomes in cancer. Mol. Cancer.

[44] Gurung S., Perocheau D., Touramanidou L., Baruteau J. (2021). The exosome journey: From biogenesis to uptake and intracellular signalling. Cell Commun. Signal..

[45] Zhang Y., Liu Y., Liu H., Tang W.H. (2019). Exosomes: Biogenesis, biologic function and clinical potential. Cell Biosci..

[46] Catalano M., O’Driscoll L. (2020). Inhibiting extracellular vesicles formation and release: A review of EV inhibitors. J. Extracell. Vesicles.

[47] Essola J.M., Zhang M., Yang H., Li F., Xia B., Mavoungou J.F., Hussain A., Huang Y. (2024). Exosome regulation of immune response mechanism: Pros and cons in immunotherapy. Bioact. Mater..

[48] Colombo M., Moita C., van Niel G., Kowal J., Vigneron J., Benaroch P., Manel N., Moita L.F., Théry C., Raposo G. (2013). Analysis of ESCRT functions in exosome biogenesis, composition and secretion highlights the heterogeneity of extracellular vesicles. J. Cell Sci..

[49] Chaput N., Angevin E., Zitvogel L., Taïeb J., Schartz N.E.C., André F. (2004). Exosome-based immunotherapy. Cancer Immunol. Immunother..

[50] Yan W., Jiang S. (2020). Immune Cell-Derived Exosomes in the Cancer-Immunity Cycle. Trends Cancer.

[51] Reiners K.S., Dassler J., Coch C., von Strandmann E.P. (2014). Role of Exosomes Released by Dendritic Cells and/or by Tumor Targets: Regulation of NK Cell Plasticity. Front. Immunol..

[52] Das A., Saha P., Kalele K., Sonar S. (2024). Clinical signature of exosomal tetraspanin proteins in cancer. Clin. Transl. Discov..

[53] Horbay R., Hamraghani A., Ermini L., Holcik S., Beug S.T., Yeganeh B. (2022). Role of Ceramides and Lysosomes in Extracellular Vesicle Biogenesis, Cargo Sorting and Release. Int. J. Mol. Sci..

[54] Arya S.B., Chen S., Jordan-Javed F., Parent C.A. (2022). Ceramide-rich microdomains facilitate nuclear envelope budding for non-conventional exosome formation. Nat. Cell Biol..

[55] Jankovičová J., Sečová P., Michalková K., Antalíková J. (2020). Tetraspanins, More than Markers of Extracellular Vesicles in Reproduction. Int. J. Mol. Sci..

[56] Lu J., Li J., Liu S., Wang T., Ianni A., Bober E., Braun T., Xiang R., Yue S. (2017). Exosomal tetraspanins mediate cancer metastasis by altering host microenvironment. Oncotarget..

[57] Egea-Jimenez A.L., Zimmermann P. (2018). Phospholipase D and phosphatidic acid in the biogenesis and cargo loading of extracellular vesicles. J. Lipid Res..

[58] Wolf A., Tanguy E., Wang Q., Gasman S., Vitale N. (2022). Phospholipase D and cancer metastasis: A focus on exosomes. Adv. Biol. Regul..

[59] Schorey J.S., Cheng Y., Singh P.P., Smith V.L. (2014). Exosomes and other extracellular vesicles in host–pathogen interactions. Embo. Rep..

[60] Martinez-Arroyo O., Selma-Soriano E., Ortega A., Cortes R., Redon J. (2021). Small Rab GTPases in Intracellular Vesicle Trafficking: The Case of Rab3A/Raphillin-3A Complex in the Kidney. Int. J. Mol. Sci..

[61] Arya S.B., Collie S.P., Parent C.A. (2023). The ins-and-outs of exosome biogenesis, secretion, and internalization. Trends Cell Biol..

[62] Wei H., Chen Q., Lin L., Sha C., Li T., Liu Y., Yin X., Xu Y., Chen L., Gao W. (2021). Regulation of exosome production and cargo sorting. Int. J. Biol. Sci..

[63] Dellar E.R., Hill C., Melling G.E., Carter D.R., Baena-Lopez L.A. (2022). Unpacking extracellular vesicles: RNA cargo loading and function. J. Extracell. Biol..

[64] Huda N., Nafiujjaman, Deaguero I.G., Okonkwo J., Hill M.L., Kim T. (2021). Nurunnabi Potential Use of Exosomes as Diagnostic Biomarkers and in Targeted Drug Delivery: Progress in Clinical and Preclinical Applications. ACS Biomater. Sci. Eng..

[65] Raposo G., Stoorvogel W. (2013). Extracellular vesicles: Exosomes, microvesicles, and friends. J. Cell Biol..

[66] Michael A., Bajracharya S.D., Yuen P.S.T., Zhou H., Star R.A., Illei G.G., Alevizos I. (2010). Exosomes from human saliva as a source of microRNA biomarkers. Oral Dis..

[67] Yakubovich E.I., Polischouk A.G., Evtushenko V.I. (2022). Principles and Problems of Exosome Isolation from Biological Fluids. Biochem. (Moscow) Suppl. Ser. A Membr. Cell Biol..

[68] Mirgh D., Krishnan A., Gorai S. (2023). Sweat exosomes: A new horizon of liquid biopsy in cancer. J. Liq. Biopsy.

[69] Daily A., Ravishankar P., Harms S., Klimberg V.S. (2022). Using tears as a non-invasive source for early detection of breast cancer. PLoS ONE.

[70] Zhao B., Lin H., Jiang X., Li W., Gao Y., Li M., Yu Y., Chen N., Gao J. (2024). Exosome-like nanoparticles derived from fruits, vegetables, and herbs: Innovative strategies of therapeutic and drug delivery. Theranostics.

[71] Zhao Y., Liu T., Zhou M. (2022). Immune-Cell-Derived Exosomes for Cancer Therapy. Mol. Pharm..

[72] Lin Z., Wu Y., Xu Y., Li G., Li Z., Liu T. (2022). Mesenchymal stem cell-derived exosomes in cancer therapy resistance: Recent advances and therapeutic potential. Mol. Cancer.

[73] Sun W., Luo J.-D., Jiang H., Duan D.D. (2018). Tumor exosomes: A double-edged sword in cancer therapy. Acta Pharmacol. Sin..

[74] Lin J., Li J., Huang B., Liu J., Chen X., Chen X.-M., Xu Y.-M., Huang L.-F., Wang X.-Z. (2015). Exosomes: Novel Biomarkers for Clinical Diagnosis. Sci. World J..

[75] Thomas S.C., Kim J.-W., Pauletti G.M., Hassett D.J., Kotagiri N. (2022). Exosomes: Biological Pharmaceutical Nanovectors for Theranostics. Front. Bioeng. Biotechnol..

[76] Wang J., Huang X., Xie J., Han Y., Huang Y., Zhang H. (2021). Exosomal analysis: Advances in biosensor technology. Clin. Chim. Acta.

[77] Janouskova O., Herma R., Semeradtova A., Poustka D., Liegertova M., Malinska H.A., Maly J. (2022). Conventional and Nonconventional Sources of Exosomes–Isolation Methods and Influence on Their Downstream Biomedical Application. Front. Mol. Biosci..

[78] Gao J., Li A., Hu J., Feng L., Liu L., Shen Z. (2023). Recent developments in isolating methods for exosomes. Front. Bioeng. Biotechnol..

[79] Bari S.M.I., Hossain F.B., Nestorova G.G. (2021). Advances in Biosensors Technology for Detection and Characterization of Extracellular Vesicles. Sensors.

[80] Cui L., Song Y., Hou Z., Yang L., Guo S., Wang C. (2025). From bench to bedside: The research status and application opportunity of extracellular vesicles and their engineering strategies in the treatment of skin defects. J. Nanobiotechnol..

[81] Chou C.-Y., Chiang P.-C., Li C.-C., Chang J.-W., Lu P.-H., Hsu W.-F., Chang L.-C., Hsu J.-L., Wu M.-S., Wo A.M. (2024). Improving the Purity of Extracellular Vesicles by Removal of Lipoproteins from Size Exclusion Chromatography- and Ultracentrifugation-Processed Samples Using Glycosaminoglycan-Functionalized Magnetic Beads. ACS Appl. Mater. Interfaces.

[82] Purushothaman A. (2019). Exosomes from Cell Culture-Conditioned Medium: Isolation by Ultracentrifugation and Characterization. The Extracellular Matrix.

[83] Faur C.I., Rotaru H., Osan C., Jurj A., Roman R.C., Moldovan M., Chirila M., Hedesiu M. (2021). Salivary exosomal microRNAs as biomarkers for head and neck cancer detection—A literature review. Maxillofac. Plast. Reconstr. Surg..

[84] Yu L.-L., Zhu J., Liu J.-X., Jiang F., Ni W.-K., Qu L.-S., Ni R.-Z., Lu C.-H., Xiao M.-B. (2018). A Comparison of Traditional and Novel Methods for the Separation of Exosomes from Human Samples. BioMed Res. Int..

[85] Guo M., Yin Z., Chen F., Lei P. (2020). Mesenchymal stem cell-derived exosome: A promising alternative in the therapy of Alzheimer’s disease. Alzheimer’s Res. Ther..

[86] Contreras H., Alarcón-Zapata P., Nova-Lamperti E., Ormazabal V., Varas-Godoy M., Salomon C., Zuniga F.A. (2023). Comparative study of size exclusion chromatography for isolation of small extracellular vesicle from cell-conditioned media, plasma, urine, and saliva. Front. Nanotechnol..

[87] Gámez-Valero A., Monguió-Tortajada M., Carreras-Planella L., La Franquesa M., Beyer K., Borràs F.E. (2016). Size-Exclusion Chromatography-based isolation minimally alters Extracellular Vesicles’ characteristics compared to precipitating agents. Sci. Rep..

[88] Guru K.T.P., Sreeja J.S., Dharmapal D., Sengupta S., Basu P.K. (2022). Novel Gold Nanoparticle-Based Quick Small-Exosome Isolation Technique from Serum Sample at a Low Centrifugal Force. Nanomaterials.

[89] Kim J., Lee H., Park K., Shin S. (2020). Rapid and Efficient Isolation of Exosomes by Clustering and Scattering. J. Clin. Med..

[90] Van Deun J., Mestdagh P., Sormunen R., Cocquyt V., Vermaelen K., Vandesompele J., Bracke M., De Wever O., Hendrix A. (2014). The impact of disparate isolation methods for extracellular vesicles on downstream RNA profiling. J. Extracell. Vesicles.

[91] Meng Y., Zhang Y., Bühler M., Wang S., Asghari M., Stürchler A., Mateescu B., Weiss T., Stavrakis S., Demello A.J. (2023). Direct isolation of small extracellular vesicles from human blood using viscoelastic microfluidics. Sci. Adv..

[92] Chernyshev V.S., Chuprov-Netochin R.N., Tsydenzhapova E., Svirshchevskaya E.V., Poltavtseva R.A., Merdalimova A., Yashchenok A., Keshelava A., Sorokin K., Keshelava V. (2022). Asymmetric depth-filtration: A versatile and scalable method for high-yield isolation of extracellular vesicles with low contamination. J. Extracell. Vesicles.

[93] Ding L., Yang X., Gao Z., Effah C.Y., Zhang X., Wu Y., Qu L. (2021). A Holistic Review of the State-of-the-Art Microfluidics for Exosome Separation: An Overview of the Current Status, Existing Obstacles, and Future Outlook. Small.

[94] Su W., Li H., Chen W., Qin J. (2019). Microfluidic strategies for label-free exosomes isolation and analysis. TrAC Trends Anal. Chem..

[95] Yang F., Liao X., Tian Y., Li G. (2017). Exosome separation using microfluidic systems: Size-based, immunoaffinity-based and dynamic methodologies. Biotechnol. J..

[96] Soares Martins T., Catita J., Martins Rosa I., A B da Cruz e Silva O., Henriques A.G. (2018). Exosome isolation from distinct biofluids using precipitation and column-based approaches. PLoS ONE.

[97] Sun Z., Yang J., Li H., Wang C., Fletcher C., Li J., Zhan Y., Du L., Wang F., Jiang Y. (2021). Progress in the research of nanomaterial-based exosome bioanalysis and exosome-based nanomaterials tumor therapy. Biomaterials.

[98] Ma X., Hao Y., Liu L. (2021). Progress in Nanomaterials-Based Optical and Electrochemical Methods for the Assays of Exosomes. Int. J. Nanomed..

[99] Li Y., Meng L., Li B., Li Y., Shen T., Zhao B. (2022). The Exosome Journey: From Biogenesis to Regulation and Function in Cancers. J. Oncol..

[100] Welsh J.A., Arkesteijn G.J.A., Bremer M., Cimorelli M., Dignat-George F., Giebel B., Görgens A., Hendrix A., Kuiper M., Lacroix R. (2023). A compendium of single extracellular vesicle flow cytometry. J. Extracell. Vesicles.

[101] Abhange K., King S., Peterson N., Sahai V., Cuneo K.C., Lubman D.M. (2025). The Use of dSTORM-Based Single Exosome Analysis To Study Tetraspanin Abundance in Extracellular Vesicles. ACS Omega.

[102] McNamara R.P., Zhou Y., Eason A.B., Landis J.T., Chambers M.G., Willcox S., Peterson T.A., Schouest B., Maness N.J., MacLean A.G. (2022). Imaging of surface microdomains on individual extracellular vesicles in 3-D. J. Extracell. Vesicles.

[103] Breitwieser K., Koch L.F., Tertel T., Proestler E., Burgers L.D., Lipps C., Adjaye J., Fürst R., Giebel B., Saul M.J. (2022). Detailed Characterization of Small Extracellular Vesicles from Different Cell Types Based on Tetraspanin Composition by ExoView R100 Platform. Int. J. Mol. Sci..

[104] Breyne K., Ughetto S., Rufino-Ramos D., Mahjoum S., Grandell E.A., de Almeida L.P., Breakefield X.O. (2022). Exogenous loading of extracellular vesicles, virus-like particles, and lentiviral vectors with supercharged proteins. Commun. Biol..

[105] Bandu R., Oh J.W., Kim K.P. (2019). Mass spectrometry-based proteome profiling of extracellular vesicles and their roles in cancer biology. Exp. Mol. Med..

[106] Kulkarni M., Kar R., Ghosh S., Sonar S., Mirgh D., Sivakumar I., Nayak A., Muthusamy R. (2024). Clinical Impact of Multi-omics profiling of extracellular vesicles in cancer Liquid Biopsy. J. Liq. Biopsy.

[107] Hartjes T.A., Mytnyk S., Jenster G.W., van Steijn V., van Royen M.E. (2019). Extracellular Vesicle Quantification and Characterization: Common Methods and Emerging Approaches. Bioengineering.

[108] Horibe S., Tanahashi T., Kawauchi S., Murakami Y., Rikitake Y. (2018). Mechanism of recipient cell-dependent differences in exosome uptake. BMC Cancer.

[109] Arneth B. (2019). Tumor Microenvironment. Medicina.

[110] Maheshwari S., Singh A.K., Arya R.K., Pandey D., Singh A., Datta D. (2014). Exosomes: Emerging Players of Intercellular Communication in Tumor Microenvironment. Discoveries.

[111] Maia J., Caja S., Moraes M.C.S., Couto N., Costa-Silva B. (2018). Exosome-Based Cell-Cell Communication in the Tumor Microenvironment. Front. Cell Dev. Biol..

[112] Hanahan D., Weinberg R.A. (2011). Hallmarks of cancer: The next generation. Cell.

[113] Petrova V., Annicchiarico-Petruzzelli M., Melino G., Amelio I. (2018). The hypoxic tumour microenvironment. Oncogenesis.

[114] Chen Z., Han F., Du Y., Shi H., Zhou W. (2023). Hypoxic microenvironment in cancer: Molecular mechanisms and therapeutic interventions. Signal Transduct. Target. Ther..

[115] Semenza G.L. (2012). Hypoxia-inducible factors: Mediators of cancer progression and targets for cancer therapy. Trends Pharmacol. Sci..

[116] Vaupel P. (2004). The Role of Hypoxia-Induced Factors in Tumor Progression. Oncologist.

[117] Meng W., Hao Y., He C., Li L., Zhu G. (2019). Exosome-orchestrated hypoxic tumor microenvironment. Mol. Cancer.

[118] Muz B., de la Puente P., Azab F., Azab A.K. (2015). The role of hypoxia in cancer progression, angiogenesis, metastasis, and resistance to therapy. Hypoxia.

[119] Zhou J., Schmid T., Schnitzer S., Brüne B. (2006). Tumor hypoxia and cancer progression. Cancer Lett..

[120] Chiang A.C., Massagué J. (2008). Molecular Basis of Metastasis. N. Engl. J. Med..

[121] Shao C., Yang F., Miao S., Liu W., Wang C., Shu Y., Shen H. (2018). Role of hypoxia-induced exosomes in tumor biology. Mol. Cancer.

[122] Park J.E., Tan H.S., Datta A., Lai R.C., Zhang H., Meng W., Lim S.K., Sze S.K. (2010). Hypoxic Tumor Cell Modulates Its Microenvironment to Enhance Angiogenic and Metastatic Potential by Secretion of Proteins and Exosomes. Mol. Cell. Proteom..

[123] Das A., Sonar S., Dhar R., Subramaniyan V. (2025). Exosomes in melanoma: Future potential for clinical theranostics. Pathol. Res. Pr..

[124] Gowda R., Robertson B.M., Iyer S., Barry J., Dinavahi S.S., Robertson G.P. (2020). The role of exosomes in metastasis and progression of melanoma. Cancer Treat. Rev..

[125] Hsu C.-Y., Chandramoorthy H.C., Mohammed J.S., Al-Hasnaawei S., Yaqob M., Kundlas M., Samikan K., Sahoo S., Sunori S.K., Abbas Z.A. (2025). Exosomes as key mediators in immune and cancer cell interactions: Insights in melanoma progression and therapy. Arch. Dermatol. Res..

[126] Roma-Rodrigues C., Fernandes A.R., Baptista P.V. (2014). Exosome in Tumour Microenvironment: Overview of the Crosstalk between Normal and Cancer Cells. BioMed Res. Int..

[127] Aslan C., Maralbashi S., Salari F., Kahroba H., Sigaroodi F., Kazemi T., Kharaziha P. (2019). Tumor-derived exosomes: Implication in angiogenesis and antiangiogenesis cancer therapy. J. Cell Physiol..

[128] Fasanaro P., D’ALessandra Y., Di Stefano V., Melchionna R., Romani S., Pompilio G., Capogrossi M.C., Martelli F. (2008). MicroRNA-210 Modulates Endothelial Cell Response to Hypoxia and Inhibits the Receptor Tyrosine Kinase Ligand Ephrin-A_3_ *. J. Biol. Chem..

[129] Landskroner-Eiger S., Moneke I., Sessa W.C. (2012). miRNAs as Modulators of Angiogenesis. Cold Spring Harb. Perspect. Med..

[130] Shao X., Hua S., Feng T., Ocansey D.K.W., Yin L. (2022). Hypoxia-Regulated TEXs and Tumor Progression: A Focus on Immune Evasion. Int. J. Mol. Sci..

[131] Guo W., Qiao T., Dong B., Li T., Liu Q., Xu X. (2022). The Effect of Hypoxia-Induced Exosomes on Anti-Tumor Immunity and Its Implication for Immunotherapy. Front. Immunol..

[132] Whiteside T.L. (2016). Tumor-Derived Exosomes and Their Role in Tumor-Induced Immune Suppression. Vaccines.

[133] Morrissey S.M., Zhang F., Ding C., Montoya-Durango D.E., Hu X., Yang C., Wang Z., Yuan F., Fox M., Zhang H.-G. (2021). Tumor-derived exosomes drive immunosuppressive macrophages in a pre-metastatic niche through glycolytic dominant metabolic reprogramming. Cell Metab..

[134] Li C., Zhou T., Chen J., Li R., Chen H., Luo S., Chen D., Cai C., Li W. (2022). The role of Exosomal miRNAs in cancer. J. Transl. Med..

[135] Tian X., Shen H., Li Z., Wang T., Wang S. (2019). Tumor-derived exosomes, myeloid-derived suppressor cells, and tumor microenvironment. J. Hematol. Oncol..

[136] Bhardwaj V., Ansell S.M. (2023). Modulation of T-cell function by myeloid-derived suppressor cells in hematological malignancies. Front. Cell Dev. Biol..

[137] Olejarz W., Dominiak A., Żołnierzak A., Kubiak-Tomaszewska G., Lorenc T. (2020). Tumor-Derived Exosomes in Immunosuppression and Immunotherapy. J. Immunol. Res..

[138] Whiteside T.L. (2017). The effect of tumor-derived exosomes on immune regulation and cancer immunotherapy. Future Oncol..

[139] Gulley J.L., Schlom J., Barcellos-Hoff M.H., Wang X.J., Seoane J., Audhuy F., Lan Y., Dussault I., Moustakas A. (2022). Dual inhibition of TGF-β and PD-L1: A novel approach to cancer treatment. Mol. Oncol..

[140] Stefańska K., Józkowiak M., Volponi A.A., Shibli J.A., Golkar-Narenji A., Antosik P., Bukowska D., Piotrowska-Kempisty H., Mozdziak P., Dzięgiel P. (2023). The Role of Exosomes in Human Carcinogenesis and Cancer Therapy—Recent Findings from Molecular and Clinical Research. Cells.

[141] Jiang J., Li J., Zhou X., Zhao X., Huang B., Qin Y. (2022). Exosomes Regulate the Epithelial–Mesenchymal Transition in Cancer. Front. Oncol..

[142] Yang C., Dou R., Wei C., Liu K., Shi D., Zhang C., Liu Q., Wang S., Xiong B. (2021). Tumor-derived exosomal microRNA-106b-5p activates EMT-cancer cell and M2-subtype TAM interaction to facilitate CRC metastasis. Mol. Ther..

[143] Shen Y.-Q., Sun L., Wang S.-M., Zheng X.-Y., Xu R. (2024). Exosomal integrins in tumor progression, treatment and clinical prediction (Review). Int. J. Oncol..

[144] Hoshino A., Costa-Silva B., Shen T.-L., Rodrigues G., Hashimoto A., Mark M.T., Molina H., Kohsaka S., Di Giannatale A., Ceder S. (2015). Tumour exosome integrins determine organotropic metastasis. Nature.

[145] Pan W., Miao Q., Yin W., Li X., Ye W., Zhang D., Deng L., Zhang J., Chen M. (2024). The role and clinical applications of exosomes in cancer drug resistance. Cancer Drug Resist..

[146] Steinbichler T.B., Dudás J., Skvortsov S., Ganswindt U., Riechelmann H., Skvortsova I.I. (2019). Therapy resistance mediated by exosomes. Mol. Cancer.

[147] Das A., Sonar S., Kalele K., Subramaniyan V. (2024). Fruit exosomes: A sustainable green cancer therapeutic. Sustain. Food Technol..

[148] Zhang Z., Lin F., Wu W., Jiang J., Zhang C., Qin D., Xu Z. (2024). Exosomal microRNAs in lung cancer: A narrative review. Transl. Cancer Res..

[149] Qin X., Yu S., Zhou L., Shi M., Hu Y., Xu X., Shen B., Liu S., Yan D., Feng J. (2017). Cisplatin-resistant lung cancer cell–derived exosomes increase cisplatin resistance of recipient cells in exosomal miR-100–5p-dependent manner. Int. J. Nanomed..

[150] Dong X., Bai X., Ni J., Zhang H., Duan W., Graham P., Li Y. (2020). Exosomes and breast cancer drug resistance. Cell Death Dis..

[151] Liu L., Jiang D., Bai S., Zhang X., Kang Y. (2024). Research progress of exosomes in drug resistance of breast cancer. Front. Bioeng. Biotechnol..

[152] Mishra A., Bharti P.S., Rani N., Nikolajeff F., Kumar S. (2023). A tale of exosomes and their implication in cancer. Biochim. Biophys. Acta Rev. Cancer.

[153] Wu Y., Cao Y., Chen L., Lai X., Zhang S., Wang S. (2024). Role of Exosomes in Cancer and Aptamer-Modified Exosomes as a Promising Platform for Cancer Targeted Therapy. Biol. Proced. Online.

[154] Dhar R., Kumarasamy V., Subramaniyan V. (2025). Signature of exosomes in cancer translational medicine. Int. J. Surg..

[155] Luo S., Chen J., Xu F., Chen H., Li Y., Li W. (2023). Dendritic Cell-Derived Exosomes in Cancer Immunotherapy. Pharmaceutics.

[156] André F., Chaput N., Schartz N.E., Flament C., Aubert N., Bernard J., Lemonnier F., Raposo G., Escudier B., Hsu D.H. (2004). Exosomes as potent cell-free peptide-based vaccine. I. Dendritic cell-derived exosomes transfer functional MHC class I/peptide complexes to dendritic cells. J. Immunol..

[157] Chen L., Zhang J., Huang Y., Zhang X., Zhang G., Kong S., Gao J., Zhang X., Ding B. (2025). Drug Delivery Systems Based on Dendritic-Cell-Derived Exosomes. Pharmaceutics.

[158] Shahir M., Hashemi S.M., Asadirad A., Varahram M., Kazempour-Dizaji M., Folkerts G., Garssen J., Adcock I., Mortaz E. (2020). Effect of mesenchymal stem cell-derived exosomes on the induction of mouse tolerogenic dendritic cells. J. Cell. Physiol..

[159] Aldahlawi A.M., Abdullah S.T. (2021). Dendritic Cell-Based Immunotherapies and their Potential use in Colorectal Cancer Immunotherapy. J. Microsc. Ultrastruct..

[160] Ghorbaninezhad F., Alemohammad H., Najafzadeh B., Masoumi J., Shadbad M.A., Shahpouri M., Saeedi H., Rahbarfarzam O., Baradaran B. (2023). Dendritic cell-derived exosomes: A new horizon in personalized cancer immunotherapy?. Cancer Lett..

[161] Dutta A. (2020). Exosomes-based cell-free cancer therapy: A novel strategy for targeted therapy. Immunol. Med..

[162] Wei B., Huang H., Cao Q., Song X., Zhang Z. (2024). Bibliometric and visualized analysis of the applications of exosomes based drug delivery. Biomed. Pharmacother..

[163] Piao Y.J., Kim H.S., Moon W.K. (2019). Noninvasive Photoacoustic Imaging of Dendritic Cell Stimulated with Tumor Cell-Derived Exosome. Mol. Imaging Biol..

[164] Rehman F.U., Liu Y., Zheng M., Shi B. (2022). Exosomes based strategies for brain drug delivery. Biomaterials.

[165] Xi X.-M., Chen M., Xia S.-J., Lu R. (2021). Drug loading techniques for exosome-based drug delivery systems. Pharmazie.

[166] Kumar D.N., Chaudhuri A., Kumar D., Singh S., Agrawal A.K. (2023). Impact of the Drug Loading Method on the Drug Distribution and Biological Efficacy of Exosomes. AAPS Pharmscitech.

[167] Morse M.A., Garst J., Osada T., Khan S., Hobeika A., Clay T.M., Valente N., Shreeniwas R., Sutton M.A., Delcayre A. (2005). A phase I study of dexosome immunotherapy in patients with advanced non-small cell lung cancer. J. Transl. Med..

[168] Yu S., Sha H., Qin X., Chen Y., Li X., Shi M., Feng J. (2020). EGFR E746-A750 deletion in lung cancer represses antitumor immunity through the exosome-mediated inhibition of dendritic cells. Oncogene.

[169] Lu Z., Zuo B., Jing R., Gao X., Rao Q., Liu Z., Qi H., Guo H., Yin H. (2017). Dendritic cell-derived exosomes elicit tumor regression in autochthonous hepatocellular carcinoma mouse models. J. Hepatol..

[170] Schioppa T., Gaudenzi C., Zucchi G., Piserà A., Vahidi Y., Tiberio L., Sozzani S., Del Prete A., Bosisio D., Salvi V. (2024). Extracellular vesicles at the crossroad between cancer progression and immunotherapy: Focus on dendritic cells. J. Transl. Med..

[171] Elsayed R., Elashiry M., Tran C., Yang T., Carroll A., Liu Y., Hamrick M., Cutler C.W. (2023). Engineered Human Dendritic Cell Exosomes as Effective Delivery System for Immune Modulation. Int. J. Mol. Sci..

[172] Barnwal A., Gaur V., Sengupta A., Tyagi W., Das S., Bhattacharyya J. (2023). Tumor Antigen-Primed Dendritic Cell-Derived Exosome Synergizes with Colony Stimulating Factor-1 Receptor Inhibitor by Modulating the Tumor Microenvironment and Systemic Immunity. ACS Biomater. Sci. Eng..

[173] Tuluwengjiang G., Rasulova I., Ahmed S., Kiasari B.A., Sârbu I., Ciongradi C.I., Omar T.M., Hussain F., Jawad M.J., Castillo-Acobo R.Y. (2024). Dendritic cell-derived exosomes (Dex): Underlying the role of exosomes derived from diverse DC subtypes in cancer pathogenesis. Pathol. Res. Pr..

[174] Al-Hawary S.I.S., Almajidi Y.Q., Bansal P., Ahmad I., Kaur H., Hjazi A., Deorari M., Zwamel A.H., Hamzah H.F., Mohammed B.A. (2024). Dendritic cell-derived exosome (DEX) therapy for digestive system cancers: Recent advances and future prospect. Pathol. Res. Pr..

[175] Wang Y., Guo X., Qin J., Xue Y., Zhang P., Liu Y., Chen M., Zhu G., Song X., Cheng L. (2025). Locoregional Immune Checkpoint Blockade and Remodeling of Lymph Nodes by Engineered Dendritic Cell-Derived Exosomes for Suppressing Tumor Progression and Metastasis. Adv. Sci..

[176] Liu Z., Pu Y., Bao Y., He S. (2021). Investigation of Potential Molecular Biomarkers for Diagnosis and Prognosis of AFP-Negative HCC. Int. J. Gen. Med..

[177] Chen T., Dai X., Dai J., Ding C., Zhang Z., Lin Z., Hu J., Lu M., Wang Z., Qi Y. (2020). AFP promotes HCC progression by suppressing the HuR-mediated Fas/FADD apoptotic pathway. Cell Death Dis..

[178] Shi S., Wang L., Wang C., Xu J., Niu Z. (2021). Serum-derived exosomes function as tumor antigens in patients with advanced hepatocellular carcinoma. Mol. Immunol..

[179] Chang C., Pei Y., Zhang C., Zhang W., Qin Y., Shi S. (2023). Combination therapy with dendritic cell loaded-exosomes supplemented with PD-1 inhibition at different time points have superior antitumor effect in hepatocellular carcinoma. Cancer Immunol. Immunother..

[180] Escudier B., Dorval T., Chaput N., André F., Caby M.-P., Novault S., Flament C., Leboulaire C., Borg C., Amigorena S. (2005). Vaccination of metastatic melanoma patients with autologous dendritic cell (DC) derived-exosomes: Results of thefirst phase I clinical trial. J. Transl. Med..

[181] Morales R.-T.T., Ko J. (2022). Future of Digital Assays to Resolve Clinical Heterogeneity of Single Extracellular Vesicles. ACS Nano.

[182] Koh H.B., Kim H.J., Kang S.-W., Yoo T.-H. (2023). Exosome-Based Drug Delivery: Translation from Bench to Clinic. Pharmaceutics.

[183] Dhar R., Devi A., Patil S., Tovani-Palone M.R. (2023). Exosomes in cancer therapy: Advances and current challenges. Electron J Gen Med..

[184] Kashkoulinejad Kouhi T. (2025). Exosome-mediated communication between T cells and dendritic cells: Implications for therapeutic strategies. Cytokine.

[185] Hazrati A., Soudi S., Malekpour K., Mahmoudi M., Rahimi A., Hashemi S.M., Varma R.S. (2022). Immune cells-derived exosomes function as a double-edged sword: Role in disease progression and their therapeutic applications. Biomark Res..

[186] Fang X., Wang Y., Wang S., Liu B. (2022). Nanomaterials assisted exosomes isolation and analysis towards liquid biopsy. Mater. Today Bio.

[187] Shao H., Im H., Castro C.M., Breakefield X., Weissleder R., Lee H. (2018). New Technologies for Analysis of Extracellular Vesicles. Chem. Rev..

[188] Yang X., Chen J., Wang N., Liu Z., Li Y. (2018). Clinical use of dendritic cell-derived exosomes for hepatocellular carcinoma immunotherapy: How far we are?. J. Hepatol..

[189] Chen J., Tan Q., Yang Z., Chen W., Zhou E., Li M., Deng J., Wu Y., Liu J., Xu J. (2025). Dendritic Cell Derived-Extracellular Vesicles Engineered to Express Interleukin-12 and Anti-CTLA-4 on Their Surface for Combinational Cancer Immunotherapy. J. Extracell. Vesicles.

